# Supplementary material for: Chromothripsis during telomere crisis is independent of NHEJ, and consistent with a replicative origin
Source: Genome Res. 2019 May;29(5):737–49. doi: 10.1101/gr.240705.118 (PMC6499312; doi:10.1101/gr.240705.118)
Supplement: Supplemental Material [file supp_gr.240705.118_Supplemental_file_1.zip › contigs/annotated_contigs/DB50/contig.2.DB50_length_752_mean_cov_9.91090425532.docx]

**DB50_length_752_mean_cov_9.91090425532**

```
CCTTGGTGTTGTTCCAGCCTGAAAACCCAAGATTTGGGGTGTTATCTGCGACCTGACCCTCCACGAGCTGAAGGCTGCTGCGGAGGGGT
 >chr10:1492981-1493414 + E=1e-236
GGGAAAGTGGTGACTTTGGGGTGACCCCACTGGCCCTCACCATAGCTGGGCACAGCACACAGTCAGATGTGCTGTCAGGGAGCTCCTGC

GAATAGGCACAGCAGCCTTGAGAAAAGCAGAGGGAGCTGATGGTGTCTGCTGTGTGCAAGCCAGAAACACAGAGAGATGTGTGTGCCAA

CAAAACCTGGAAAATAACCATGTCCTCACAGCCTGGGGACTCTGGGAAGTCCTACAGCAAAGAAGAAGCCTGGTCTGCCCATCTTTGTG

TGACAAGTGTTTGCAGGAAATAGTTCATCAAAAAACACTTTTTCTCAGGAGACAACTAGGAATCACCTAGGA|CACAC|CTTGATGGTT
                                                                         >chr10:1498517
AATTCCATCAACCTTCAAGTCCCCAAAATGGCCCCTTGCAGGATCGTTGCTGAGGCAGAGCTGGGTTCACGAGACCCCCTGGAGAGAGG
-1498841 - E=4e-174
TGGGGGTTGCAGCCTGGCAAGAGCTAAGAGCAGAAAATTAGTGTGGGGTGTTCATAGGTTTTGGGCCGGGGACGAGAGGCATGAGAGCC

AAGAACTGGCTTGTATGGAGCAAAGTTAATGAGGAAACTTTAGGTTGGTAGGAAGAGCAGACGTCTTCCGGGCAGGAGCCTCCAGGAGC

CGTTGCTGGTGTTGAGAAGGAAGTGACTTGGTTGGTTGGTTC
```
